# Strategic planning to improve surgical, obstetric, anaesthesia, and trauma care in the Asia–Pacific region: introduction

**DOI:** 10.1186/s12919-023-00254-1

**Published:** 2023-07-25

**Authors:** Rennie X. Qin, Zachary G. Fowler, Sangchul Yoon, Anusha Jayaram, Makela Stankey, Salmaan Keshavjee, Annette Holian, Geoff Ibbotson, Kee B. Park

**Affiliations:** 1 The Program in Global Surgery and Social Change, the Department of Global Health and Social Medicine, Harvard Medical School, 641 Huntington Ave, Boston, MA 02115 USA; 2 Department of Medical Humanities and Social Sciences, College of Medicine, Yonsei University, Seoul, South Korea; 3 Tufts University School of Medicine, 145 Harrison Ave, Boston, MA 02111 USA; 4 Keck School of Medicine at the University of Southern California, 1975 Zonal Ave, Los Angeles, CA 90033 USA; 5 Center for Global Health Delivery, the Department of Global Health and Social Medicine, Harvard Medical School, 641 Huntington Ave, Boston, MA 02115 USA; 6 Royal Australasian College of Surgeons, 250-290 Spring Street, East Melbourne, VIC 3002 Australia; 7 United Nations Institute for Training and Research (UNITAR), Palais Des Nations, 1211 Geneva 10, Switzerland; 8 The Global Surgery Foundation, Rue Rodolphe-Toepffer 11 Bis C/O Altenburger Ltd, 1206 , Genève 10, Switzerland

**Keywords:** Global surgery, Surgical system strengthening, National surgical planning, Asia–Pacific

## Abstract

Surgical, obstetric, and anaesthesia care are required to treat one-third of the global disease burden. They have been recognised as an integral component of universal health coverage. However, five billion people lack access to safe and affordable surgical care when required. Countries in the Asia–Pacific region are currently developing strategies to strengthen their surgical care systems. The *Strategic Planning to Improve Surgical, Obstetric, Anaesthesia, and Trauma Care in the Asia–Pacific Region* meeting is a three-part virtual meeting series that brought together Ministries of Health, intergovernmental organisers, funders, professional associations, academic institutions, and nongovernmental organisations in the Asia–Pacific region. The meeting series took place over three virtual sessions in February and March 2021. Each session featured framing talks, panel presentations, and open discussions. Participants shared lessons about the challenges and solutions in surgical system strengthening, discussed funding opportunities, and forged strategic partnerships. Participants discussed strategies to build ongoing political momentum and mobilise funding, the implications of the COVID-19 pandemic and climate change on surgical care, the need to build a broad-based, inclusive movement, and leveraging remote technologies for workforce development and service delivery. This virtual meeting series is only the beginning of an ongoing community for knowledge sharing and strategic collaboration towards surgical system strengthening in the Asia–Pacific region.

## Background 

**Fig. 1:**
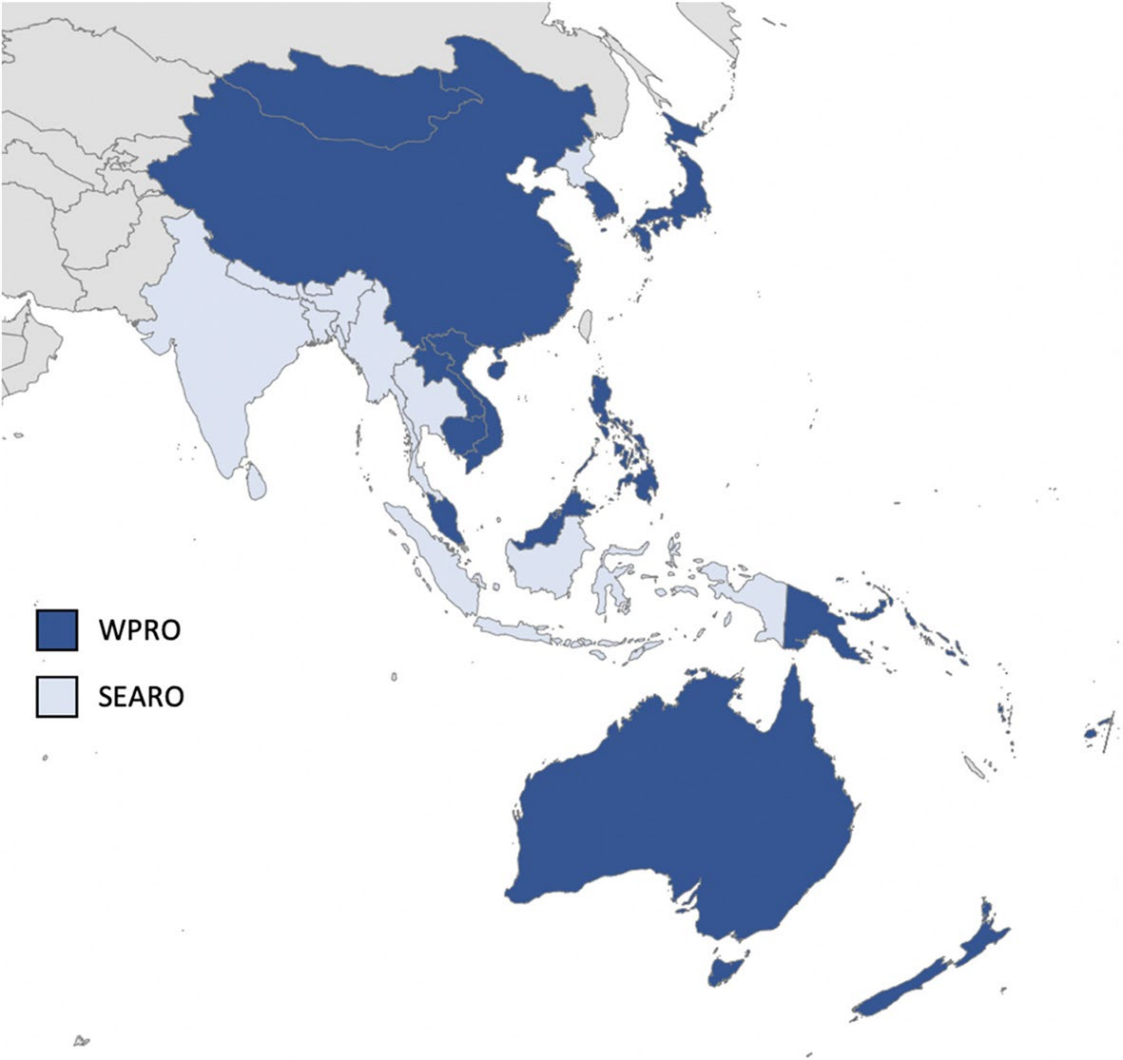
Map of the Asia–Pacific region

Surgical, obstetric, and anaesthesia care is a critical component of universal health coverage (UHC). However, five billion people lack access to safe, timely, and affordable surgical care globally, nine out of ten of them live in low- and middle-income countries (LMICs) [1]. In 2015, the Lancet Commission on Global Surgery (LCoGS) recommended the development of National Surgical, Obstetric, and Anaesthesia plans (NSOAPs) to strengthen surgical care using a systems-based approach [1]. Assessment of surgical care capacity in the Asia–Pacific region revealed challenges in access, affordability, service volume, and workforce density [2]. In 2019, Harvard Medical School Center for Global Health Delivery (HMS CGHD) and the Program in Global Surgery and Social Change (PGSSC) hosted the *High-Level National Surgical, Obstetric, and Anaesthesia Planning Meeting* in Dubai. It brought together 77 global, regional, and country authorities and funders. Since that time, strategic planning to improve surgical care has been underway in several countries in the Asia–Pacific region* (Fig. 1), with many others expressing interest or commitment. In 2019, Pacific Health Ministers at their thirteenth meeting championed a pacific-specific approach to developing and implementing NSOAPs towards universal health coverage (UHC) and the Healthy Vision [3]. At the 71^st^ session of the World Health Organization (WHO) Regional Committee for the Western Pacific in 2020, Member States adopted the *Action Framework for Safe and Affordable Surgery in the Western Pacific Region (2021–2030),* which provides a roadmap for improving access, quality, and affordability of surgery in the region [2].

## Meeting planning

In February and March 2021, a series of three virtual meetings were convened to engage a broad group of stakeholders to lead initiatives to strengthen surgical, obstetric, and anaesthesia care in the Asia–Pacific region (‘surgical care’) ** (Panel 1). This meeting series was co-hosted by the HMS PGSSC and CGHD, the United Nations Institute for Training and Research (UNITAR), the Global Surgery Foundation, and the Royal Australasian College of Surgeons (RACS).

Previous high-level meetings on national surgical planning had been held in person. However, due to the COVID-19 pandemic, we pivoted to a virtual format. We broke down a two-day in-person meeting into three fortnightly spaced sessions to maintain engagement and avoid zoom fatigue. The topics of the three sessions were:

- Current status and opportunities in surgical system strengthening (18.^th^ February)
- Financing surgical, obstetric, anaesthesia, and trauma care (4.^th^ March)
- Strategic partnerships in supporting surgical care (18.^th^ March)

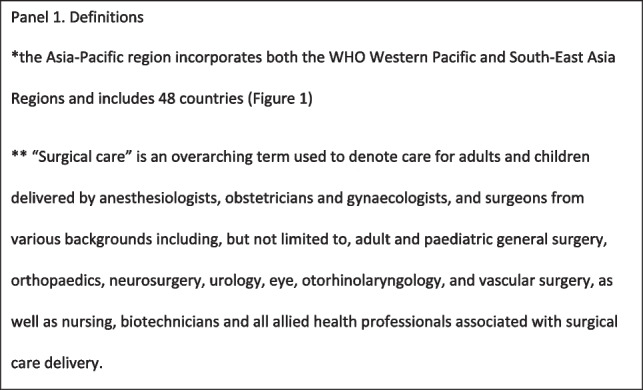


## Meeting objectives

The objectives of the meeting series were to:

- Provide recent updates and historical context for surgical capacity strengthening in the Asia-Pacific region and globally;
- Identify challenges and opportunities to improve surgical care in the Asia-Pacific region;
- Discuss the role of partnerships with the World Health Organization, funding agencies, professional associations, academic institutions, and private organisations in support of strategic planning and implementation of surgical care; and
- Explore the possibilities for coordinated efforts and regional collaboration.

## Participation

This virtual meeting series was primarily aimed at high-level global, regional, and country authorities and funders in the Asia–Pacific Region. The target audience included Ministries of Health, funding agencies, intergovernmental organisations, professional societies, academic institutions, and the private sector.

Leveraging on the virtual format, the meeting series was also made openly viewable in a webinar format to encourage civil society participation and engagement. Advertisements to a public audience occurred through partner organisations’ mailing lists and social media networks.

High-level participants were able to interact with each other through audio and video. Webinar attendees could interact with the meeting participants through typed messages. They could also be promoted to participants with audio and video capabilities when they enter a discussion.

101 high-level participants representing five Ministries of Health, two WHO regions, eight funding agencies, professional societies, academic institutions, nongovernmental organisations, and the private sector attended the three sessions. 467 public attendees from 72 countries registered to view the meeting in a webinar format.

## Conference program

Each session commenced with framing talks, followed by panel presentations and discussions. Table 1 presents a summary of the content, speakers, and attendance of each session.

**Table 1:** An overview of meeting content, speakers, and participation

Session title	Moderators	Framing Talks	Panelists	Number Attended
**Session 1** Current status of surgical care in the Asia–Pacific region and opportunities for improvement	Dr Kee Park Dr Liz McCleod	Dr John G Meara Dr Takeshi Kasai Dr Poonam Singh Dr Nikhil Seth Dr Elizabeth McLeod	Dr Howard Sobel (WHO WPRO) Lord Viliami Tangi (Tonga) Dr Mohamed Yusof bin Abdul Wahab (Malaysia) Hon Dr Ifereimi Waqainabete (Fiji) Dr Aumea Herman (Cook Islands) Dr Bikash Devkota (Nepal)	223 from 34 countries
**Session 2** Financing for surgical, obstetric, anaesthesia, and trauma care in the Western Pacific Region	Dr Lubna Samad Dr Kiki Maoate	Dr Jim Kim Dr Blake Alkire Prof Rifat Atun Dr Muhammad Ali Pate	Dr Peter Cowley (WHO WPRO) Dr Ammar Abdo Ahmed (IsDB) Ms Peggy Tse (IFC) Dr Do Hyeong Kim (KOICA Prof Tanaka Go (JICA) Dr Monique Wubbenhorst (USAID) Dr Anil Shrestha (Nick Simons Institute) Mr Shanthakumar Bannirchelvam (Global Impact Partners)	215 from 42 countries
**Session 3** Strategic partnerships to improve surgical care in the Asia–Pacific region	Dr Adrian Gelb Prof David Watters	Dr Paul Farmer Dr Berlin Kafoa Dr Annette Holian	Dr Geoff Ibbotson (UNITAR) Dr Wayne Morriss (WFSA) Dr Amanda Noovao-Hill (FNU/RANZCOG) Dr Rebecca Mitchell (RANZCOG) Prof Tahmina Banu (GICS) Prof Sergelen Orgoi (Mongolia WHO CC) Dr Nobhojit Roy (Mumbai WHO CC) Dr William May (FNU) Dr Michael McGlynn (Interplast) Prof Annie Cheung (Lancet Commission on Diagnostics) Dr Richard Henker (American Association of Nurse Anesthetists) Mr Ashish Kohli (Johnson & Johnson)	161 from 32 countries

## A summary of the meeting proceedings

Across all three sessions, there were lively discussions among participants through both verbal and written communication channels. Several themes emerged.

- **Building political momentum**: though substantial progress has been made since 2015, participants agreed that there is still an ongoing need to educate funders and policymakers about the indispensable role of surgical care within UHC. Advocacy to generate political momentum and funding should align surgical care with other health priorities, such as health security and maternal and child health.
- **COVID-19 pandemic:** whilst the COVID-19 pandemic has constricted fiscal space for surgery, it has highlighted the contribution of surgical care systems to pandemic preparedness and health security. Short-term interest in building critical care capacity must be leveraged to generate long-term investment in surgical system strengthening towards health system resilience.
- **Stakeholder representation and engagement**: participants called for the movement to improve surgical care to be inclusive of not only obstetrics and anaesthesia but also other disciplines that form the broader ecosystem around surgical care, such as emergency medicine and diagnostics. Not only do specialists play an important role but also nurses, midwives, trainees, and students. Improving surgical care requires using imagination to forge collaborations beyond the health sector, for example, with environmental management and trade. Limited patient and civil society engagement, thus far, is a major obstacle to the global surgery movement. Future efforts in national surgical planning must include the voices of patients and citizens, especially those from marginalised communities.
- **Implementation, monitoring, and evaluations of NSOAPs:** participants urged that strong partnerships must be sustained into the NSOAP implementation stage, given that NSOAP implementation can present more challenges and complexities than their development. Aside from funding, another challenge in NSOAP implementation is monitoring and evaluation. Participants agreed that progress in surgical system strengthening must be monitored through a set of standard key performance indicators LCoGS indicators. In the Pacific region, four LCoGS indicators have been collected by 13 countries; [4] Malaysia has collected three LCoGS indicators and maintains a national obstetric registry.
- **Leveraging remote technology:** the COVID-19 pandemic has also provided an opportunity to leverage remote technology for surgical care. Remote technology could play a role in both workforce strengthening and service delivery. In workforce strengthening, virtual platforms could be used to develop online training modules, peer reviews, and complex case discussions. In service delivery, it could be employed in telemedicine for hard-to-reach populations. Participants identified remote technology as a strategy that could potentially generate quick wins and improve the visibility of surgical system strengthening in the Asia–Pacific region.
- **Climate change:** participants recognised climate change as an important regional and global challenge that needs to be incorporated into strategic planning for surgical care. They illustrated several areas of overlap between climate change and surgical system strengthening, including climate-resilient surgical infrastructure; water, sanitation and hygiene services; infection prevention and control and emergency-ready mobile surgical platforms.
- **A plurality of surgical system strengthening efforts:** participants recognised that aside from developing comprehensive, overarching NSOAPs, there is a role for diagonal, facility-based initiatives that could generate quick results and heighten the visibility of surgical system strengthening. There can be a plurality of initiatives in surgical system strengthening, both comprehensive and focused, large and small, outlined throughout these proceedings, that could advance safe and affordable surgery in the Asia–Pacific region.

## Post-meeting survey and follow-up

A survey was distributed to participants after the meeting series. Most participants agreed that the meeting series achieved its objectives. 81.5% of participants agreed or strongly agreed that the meeting furthered their understanding of surgical system strengthening. 76.2% of participants agreed or strongly agreed that the meeting facilitated partnership building. We assessed whether the format of the meeting series was conducive to partnership building using a five-point Likert scale, with 1 being very inconducive and 5 being very conducive. The mean rating was 4.4 with a standard deviation of 0.8.

132 participants agreed to share their contact details with other participants of this meeting series. We distributed a post-conference booklet containing contact details to facilitate ongoing networking and partnership building. In terms of follow-up action, 76 participants would like letter templates to WHO regional offices, 75 would like emails connecting them to other meeting attendees, 66 would like a letter template to their Ministries of Health, 63 would like a letter template to academic partners, 59 would like a letter templates to funder, 55 would like a letter template to professional associations, and 26 would like other letter templates.

## Strengths and Limitations

The strength of this meeting series is the number of high-level participants represented across Ministries of Health, intergovernmental organisations, funders, professional associations, and academic institutions. Due to the COVID-19 pandemic, we had to adapt the meeting to a virtual format. However, this allowed the high-level meeting to be more inclusive. Without travel and visa restrictions, many participants from LMICs were able to attend. We broadcasted the previously closed high-level meeting to a public audience in a webinar format. This resonates with the importance of building a broad-based and inclusive global surgery movement at the grassroots level and not only at a high level. Despite the virtual format, there were ample verbal and written discussions between participants throughout the meeting series. However, the virtual meeting series was limited in replicating the networking experience of in-person meetings. Collaborations and partnerships often spontaneously form in social settings at in-person conferences. Due to considerations of zoom fatigue, we had to reduce the sessions’ duration, which limited the potential for in-depth technical sharing. The virtual nature of the meeting series also posed challenges with scheduling. Inevitable, participants from one part of the world will be unable to join live due to time zone differences. We made a conscious decision to prioritise time zones in the Asia–Pacific region, which meant that participants from Europe, Africa, and parts of the United States had to make recorded presentations and could not participate in live discussions. Despite efforts to ensure equal representation across gender, region, and professional background, participant representation at this meeting remains skewed. There were many participants from the Pacific region, which reflects the amount of surgical system strengthening activities currently occurring in the region. However, organisations and personnel from the United States were also over-represented, likely due to the conference organisers’ professional networks. Future regional meetings should reflect on whether the meeting objective is to facilitate regionalism: collaboration between countries within a region towards a common purpose or regionalisation: when external countries and organisations work in collaboration with a region [5]. Moreover, future meetings should incorporate patients and service users’ perspective and further explore the social determinants of health encountered.

We found that virtual meetings have a utility beyond the COVID-19 pandemic in increasing inclusivity and reducing the cost, environmental impact, and barriers associated with international travel.

## Conclusion

By the end of the meeting series, participants were prepared to:

- Support, advocate for, and undertake strategic planning to improve surgical care in the Asia–Pacific region
- Build strategic partnerships with national, regional, and international stakeholders towards surgical system strengthening.
- Frame financing of surgical care as indispensable to universal health coverage (UHC), pandemic preparedness, and economic development

## Recommendations

- Countries to develop strategic plans to strengthen their surgical systems through a broad system-based approach, contextualised to their national circumstances.
- Track and report progress in surgical system strengthening according to key performance indicators set out in monitoring and evaluation frameworks.
- WHO Regional and Country Offices to provide technical support to countries in surgical system strengthening. WHO SEARO to develop dedicated structures or frameworks to support surgical system strengthening, similar to the WHO WPRO *Action Framework*.
- Ongoing education, advocacy, and lobbying of funders and policymakers on the indispensable role of surgical care within UHC.
- Countries and funders to mobilise domestic and international funding sources for surgical system strengthening, including catalytic funding mechanisms.
- Leverage funding opportunities generated by the COVID-19 pandemic to build sustainable long-term programs for surgical system strengthening.
- Include representatives from surgery, anaesthesia, obstetrics and gynaecology, and other specialties in the ecosystem around surgical care on steering committees and stakeholder consultation meetings for national surgical planning.
- Engage civil society in all efforts to strengthen surgical systems, including but not limited to advocacy and awareness-raising events, stakeholder consultation for surgical strategic planning, and community participation in global surgery research.
- Explore high-impact strategies such as using remote technology and simulation in training, workforce strengthening, and service delivery.
- Create a community for ongoing technical sharing, partnership building, and regional collaboration in surgical system strengthening in the Asia–Pacific region.

## Funding 

This meeting and publication were funded by the Harvard Medical School Center for Global Health Delivery. The funders had no role in preparation of the meeting content or meeting report.

## Data Availability

N/A.

## Abbreviations

CGHD: Center for Global Health Delivery
DFAT: Department of Foreign Affairs and Trade
GICS: Global Initiative for Children’s Surgery
FNU: Fiji National University
HMS: Harvard Medical School
IFC: International Finance Corporation
IsDB: Islamic Development Bank
JICA: Japan International Cooperation Agency
KOICA: Korea International Cooperation Agency
LCoGS: Lancet Commission on Global Surgery
LMICs: Low- and middle-income countries
NSOAP: National Surgical, Obstetric, and Anaesthesia Plan
PGSSC: Program in Global Surgery and Social Change
RACS: Royal Australasian College of Surgeons
RANZCOG: Royal Australian and New Zealand College of Obstetricians and Gynaecologists
UHC: Universal health coverage
UNITAR: United Nations Institute for Training and Research
USAID: United States Agency for International Development
WFSA: World Federation of Societies of Anaesthesiologists
WHO: World Health Organization
WHO CC: World Health Organization Collaborating Center
WHO SEARO: World Health Organization South-East Asia Region
WHO WPRO: World Health Organization Western Pacific Region
